# Physicochemical Properties of Novel Copolymers of Quaternary Ammonium UDMA Analogues, Bis-GMA, and TEGDMA

**DOI:** 10.3390/ijms24021400

**Published:** 2023-01-11

**Authors:** Marta W. Chrószcz-Porębska, Izabela M. Barszczewska-Rybarek, Grzegorz Chladek

**Affiliations:** 1 Department of Physical Chemistry and Technology of Polymers, Faculty of Chemistry, Silesian University of Technology, Strzody 9 Str., 44-100 Gliwice, Poland; 2 Department of Engineering Materials and Biomaterials, Faculty of Mechanical Engineering, Silesian University of Technology, Konarskiego 18A Str., 44-100 Gliwice, Poland

**Keywords:** quaternary ammonium methacrylate, urethane-dimethacrylate analogue, dimethacrylate-based dental material, photocured copolymer, physicochemical property

## Abstract

This study aimed to elucidate the physicochemical properties of copolymers comprising 40 wt.% bisphenol A glycerolate dimethacrylate (Bis-GMA), 40 wt.% quaternary ammonium urethane-dimethacrylate analogues (QAUDMA-m, where *m* corresponds to the number of carbon atoms in the *N*-alkyl substituent), and 20 wt.% triethylene glycol dimethacrylate (TEGDMA) copolymers (BG:QAm:TEGs). The BG:QAm:TEG liquid monomer compositions and reference compositions (40 wt.% Bis-GMA, 40 wt.% urethane-dimethacrylate (UDMA), 20 wt.% TEGDMA (BG:UD:TEG) and 60 wt.% Bis-GMA, 40 wt.% TEGDMA (BG:TEG)) were characterized in terms of their refractive index (*RI*) and monomer glass transition temperature (*Tg_m_*) and then photocured. The resulting copolymers were characterized in terms of the polymer glass transition temperature (*Tg_p_*), experimental polymerization shrinkage (*S_e_*), water contact angle (*WCA*), water sorption (*WS*), and water solubility (*SL*). The prepared BG:QAm:TEG liquid monomer compositions had *RI* in the range 1.4997–1.5129, and *Tg_m_* in the range −52.22 to −42.12 °C. The BG:QAm:TEG copolymers had *Tg_p_* ranging from 42.21 to 50.81 °C, *S_e_* ranging from 5.08 to 6.40%, *WCA* ranging from 81.41 to 99.53°, *WS* ranging from 25.94 to 68.27 µg/mm^3^, and *SL* ranging from 5.15 to 5.58 µg/mm^3^. Almost all of the developed BG:QAm:TEGs fulfilled the requirements for dental materials (except BG:QA8:TEG and BG:QA10:TEG, whose *WS* values exceeded the 40 µg/mm^3^ limit).

## 1. Introduction

Over the past three decades, quaternary ammonium methacrylates (QAMs) have attracted the attention of scientists in the field of antibacterial dimethacrylate-based dental composites [1,2]. This is due to the fact that QAMs show high antibacterial activity caused by the presence of quaternary ammonium (QA) moieties in their structures. As the QA moiety contains a positively charged quaternary nitrogen atom, it can adsorb on the negatively charged bacteria cell surface. As a result, the long alkyl chain attached to the quaternary nitrogen can interact with the lipid chains in a cell wall and disturb the cell’s electric balance. This causes an increase in the bacteria cell osmotic pressure which leads to its death [3]. What is more, QAMs can be permanently embedded into composite matrices via copolymerization of their double bonds with double bonds of other dimethacrylates constituting the composite matrix [4]. Thus, QAMs may offer long-term antibacterial activity [5,6]. Various QAMs have been described in the literature, including 2-(dimethylamino)ethyl methacrylate derivatives with chloride [7,8,9], bromide [10,11,12,13,14,15], and iodide [16,17] counter ions, quaternary ammonium derivative of bisphenol A glycerolate dimethacrylate [18], and fully aliphatic [19] and cycloaliphatic [20,21] urethane-dimethacrylates. Such QAMs represent potential antibacterial components of dental composites because they offer high antibacterial activity against various strains, including *Streptococcus mutans* [4,5,10,13,14,15,18,20,21], *Staphylococcus aureus* [5,18], and *Escherichia coli* [18]. However, the well-known QAMs induce a loss of mechanical properties and an increase in the amount of water absorbed by the material and in residual fraction release [4,5,10,13,14,15,18,19,21,22]. Nevertheless, the field of antibacterial dimethacrylates has continued to evolve, and there are still many possibilities to design new compounds with chemical structures that are suitable for obtaining dental composites with appropriate physicochemical, mechanical, and antibacterial characteristics. 

**Figure 1:**
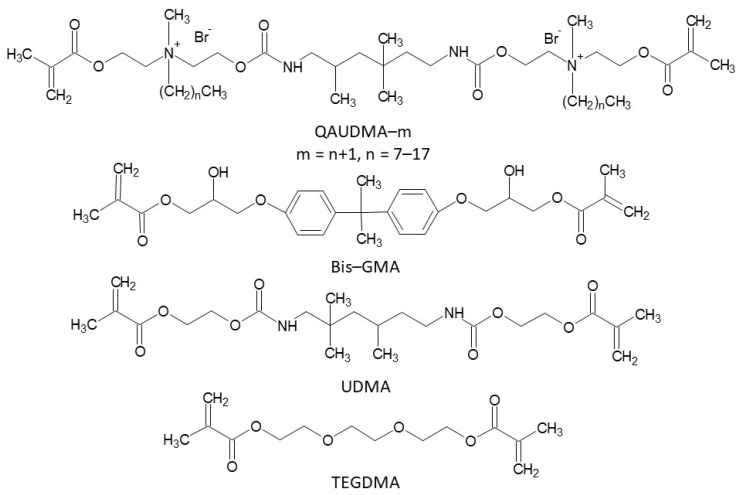
Chemical structures of dimethacrylate monomers used in this study.

This study provides insights relevant for preparing bioactive dimethacrylate matrices for dental composite restorative materials. Our proposal is based on the utilization of quaternary ammonium urethane-dimethacrylate analogues (QAUDMA-m, where *m* is number of carbon atoms in the *N*-alkyl substituent) (Figure 1) [23]. These compounds comprise a 2,2,4-trimethylhexamethylene diisocyanate (TMDI) core and two methacrylate-terminated wings. The quaternary ammonium groups, each substituted with an *N*-alkyl chain of 8, 10, 12, 14, 16, or 18 carbon atoms, are located in the middle of each wing. Because the QAUDMA-m monomers were viscous resins with suitable physicochemical parameters (refractive index (*RI*) from 1.50 to 1.52, monomer glass transition temperature (*Tg_m_*) from −31 to −15 °C, degree of conversion (*DC*) from 0.53 to 0.78, experimental polymerization shrinkage (*S_e_*) from 1.24 to 2.99%) [23], we evaluated them in the context of their copolymers with common dental dimethacrylates: bisphenol A glycerolate dimethacrylate (Bis-GMA), urethane-dimethacrylate monomer (UDMA), and triethylene glycol dimethacrylate (TEGDMA) (Figure 1).

First, the physicochemical, mechanical, and antibacterial properties of QAUDMA-m copolymers with 40 wt.% TEGDMA (QAm:TEGs) were characterized [24,25]. The uncured QAm:TEGs had appropriate transparency (*RI* from 1.4895 to 1.5001) and molecular mobility (*Tg_m_* from −33.82 to −25.84 °C). The QA:TEG copolymers were characterized by low volumetric contraction (*S_e_* from 6.4 to 6.9%), high polymerization efficiency (*DC* from 84.0 to 88.7%), high stiffness (polymer glass transition temperature (*Tg_p_*) from 60.33 to 66.32 °C), suitable surface properties (water contact angle (*WCA*) from 82.1 to 98.7°), and high antibacterial activity against *S. aureus* (number of adhered bacteria from 0 (no adhered bacteria) to 4.84 log(CFU/mL), inhibition zone from 6 to 19 mm), and *E. coli* (number of adhered bacteria from 0 (no adhered bacteria) to 3.34 log(CFU/mL), inhibition zone from 6 to 10 mm) [24]. However, they showed excessively high water sorption (*WS* from 116.08 to 148.31 µg/mm^3^) and solubility (*SL* from 12.67 to 52.39 µg/mm^3^) [25]. These properties excluded them from potential use as matrices for dental composites. 

Therefore, the present study aimed to achieve and characterize the novel copolymers comprising 40 wt.% Bis-GMA, 40 wt.% QAUDMA-m, and 20 wt.% TEGDMA (BG:QAm:TEGs). 

Proper functioning and longevity of dental restorations equally depends on the physicochemical properties and mechanical performance. The most important physicochemical properties of dental materials’ matrices include (i) *DC*—which should exceed 0.55 to provide clinical efficacy [26]; (ii) *S_e_*—which should be as low as possible, because the lower the *S_e_*_,_ the narrower the marginal gaps between the reconstruction and adjacent tooth tissue, and therefore, the smaller the area privileged for bacteria growth [27]; (iii) *Tg_p_*—which should be higher than 40 °C, ensuring that the material is in a glassy state in a temperature range occurring in the oral cavity [28]; (iv) *WS*—which should not exceed 40 µg/mm^3^ [29], because the excess of water absorbed by the material can cause its swelling and decrease its mechanical properties [30]; and (v) *SL*—which should not be higher than 7.5 µg/mm^3^ [29], because usually, the higher the *SL,* the lower the mechanical stability of the restoration [31].

Despite the crucial role of the dental composites’ physicochemical properties in governing their stability, longevity, and functionality, these aspects are rarely examined. In most cases, studies on dental composite matrices modified with QAMs are only limited to the examination of their antibacterial activity, *DC*, and mechanical properties. 

The aim of this study was the preliminary assessment of the applicability of BG:QAm:TEGs as matrices for dental dimethacrylate-based composites from the perspective of their physicochemical properties. These evaluations were based on measurements of the *RI* and *Tg_m_* of liquid monomer compositions and the *Tg_p_*, *S_e_*, theoretical polymerization shrinkage (*S_t_*), *WCA*, *WS*, and *SL* of corresponding copolymers. The *DC* [32] was also discussed. 

## 2. Results

In this study, eight compositions of dimethacrylate monomers were prepared. Six of them consisted of 40 wt.% Bis-GMA, 40 wt.% QAUDMA-m, and 20 wt.% TEGDMA (BG:QAm:TEGs). For comparison, the 40 wt.% Bis-GMA, 40 wt.% UDMA, 20 wt.% TEGDMA (BG:UD:TEG), 60 wt.% Bis-GMA, and 40 wt.% TEGDMA (BG:TEG) compositions were also prepared. Liquid monomers were characterized in terms of density (*d_m_*), *RI*, and *Tg_m_*, and the corresponding polymers were characterized in terms of density (*d_p_*), *S_t_*, *S_e_*, *Tg_p_*, *WCA*, *WS*, and *SL*.

In the BG:QAm:TEG notation, the *m* still corresponds to the number of carbon atoms in the *N*-alkyl substituent. Later, *Cm* is used to denote the length of the *N*-alkyl substituent in QAUDMA-m and BG:QAm:TEG.

### 2.1. Properties of Liquid Monomer Compositions

Table 1 summarizes the *RI* and *d_m_* values of the studied liquid monomer compositions.

The *RI* of BG:QAm:TEGs ranged from 1.4982 to 1.5129. The BG:QAm:TEGs with *Cm* of C8 and C10 had lower *RI* than BG:TEG (*RI* = 1.5048) (*p* ≤ 0.05), while BG:QAm:TEGs with *Cm* from C12 to C18 had higher *RI* (*p* ≤ 0.05). The BG:QAm:TEGs with *Cm* of C12 and C14 had *RI* values similar to that of BG:UD:TEG (*RI* = 1.5127; *p* > 0.05), whereas the *RI* values of the remaining BG:QAm:TEGs were lower than those of BG:UD:TEG (*p* ≤ 0.05). 

The *d_m_* of BG:QAm:TEGs ranged from 1.099 to 1.154 g/cm^3^ and decreased as *Cm* increased. Specifically, BG:QAm:TEGs with *Cm* from C8 to C12 had higher *d_m_* values than the reference copolymers (*d_mBG:UD:TEG_* = 1.115 g/cm^3^, *d_mBG:TEG_* = 1.102 g/cm^3^) (*p* ≤ 0.05), whereas the remaining BG:QAm:TEGs had *d_m_* values similar to the reference copolymers (*p* > 0.05).

**Figure 2:**
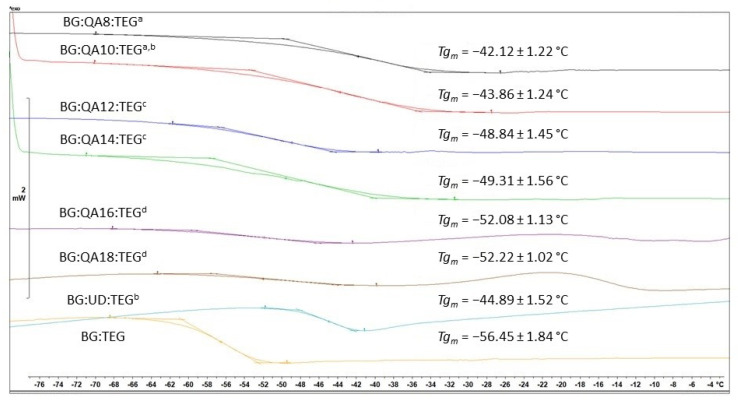
Results of DSC experiments involving the investigated liquid monomer compositions, revealing their glass transition temperatures. Lowercase letters indicate statistically insignificant differences (*p* > 0.05, non-parametric Wilcoxon test).

The *Tg_m_* of BG:QAm:TEGs ranged from −52.22 to −42.12 °C and decreased as *Cm* increased. Almost all of the investigated BG:QAm:TEGs had lower *Tg_m_* than BG:UD:TEG (*Tg_m_* = −44.89 °C) (*p* ≤ 0.05), except BG:QA10:TEG and BG:QA8:TEG. BG:QA10:TEG had a similar *Tg_m_* (*p* > 0.05), whereas BG:QA8:TEG had a higher *Tg_m_* (*p* ≤ 0.05) compared with that of BG:UD:TEG. All BG:QAm:TEGs had higher *Tg_m_* than BG:TEG (*Tg_m_* = −56.45 °C) (*p* ≤ 0.05). Representative differential scanning calorimetry (DSC) thermograms of liquid monomer compositions are shown in Figure 2.

### 2.2. Properties of Copolymers

Table 2 summarizes the *d_p_*, *S_t_*, and *S_e_* of the studied copolymers. 

The *d_p_* of BG:QAm:TEGs ranged from 1.174 to 1.216 g/cm^3^ and decreased as the *Cm* increased. BG:QA8:TEG had a higher *d_p_* than BG:TEG (*d_p_* = 1.206 g/cm^3^) (*p* ≤ 0.05), while BG:QA10:TEG and BG:QA12:TEG had *d_p_* values similar to that of BG:TEG (*p* > 0.05). The remaining BG:QAm:TEGs had *d_p_* values lower than those of BG:TEG (*p* ≤ 0.05). The BG:QAm:TEGs generally had lower *d_p_* than BG:UD:TEG (*d_p_* = 1.215 g/cm^3^) (*p* ≤ 0.05), except for BG:QA8:TEG, which had a similar *d_p_* (*p* > 0.05). 

The *S_t_* of BG:QAm:TEGs ranged from 8.90% to 9.81% and decreased as the *Cm* increased. All of the studied BG:QAm:TEGs had lower *S_t_* values compared with the reference samples (*S_tBG:UD:TEG_* = 12.90%, *S_tBG:TEG_* = 11.61%) (*p* ≤ 0.05). The opposite relationship was observed for *S_e_*. Specifically, the *S_e_* of BG:QAm:TEGs ranged from 5.08% to 6.40% and increased as the *Cm* increased. Compared with the reference samples (*S_eBG:UD:TEG_* = 8.35%, *S_eBG:TEG_* = 8.07%), all of the studied BG:QAm:TEGs had lower *S_e_* (*p* ≤ 0.05). 

**Figure 3:**
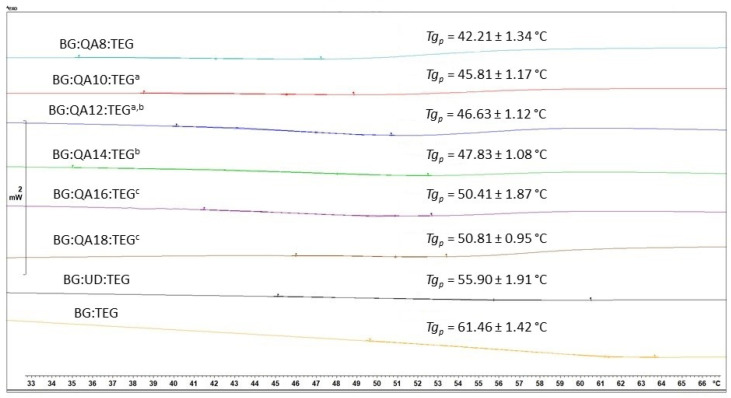
Results of DSC experiments involving the studied copolymers revealing their glass transition temperatures. Lowercase letters indicate statistically insignificant differences (*p* > 0.05, non-parametric Wilcoxon test).

The *Tg_p_* of BG:QAm:TEGs ranged from 42.21 to 50.81 °C and increased as the *Cm* increased. All of the studied BG:QAm:TEGs had lower *Tg_p_* values compared with the reference copolymers (*Tg_pBG:UD:TEG_* = 55.90 °C, *Tg_pBG:TEG_* = 61.46 °C) (*p* ≤ 0.05). Representative DSC thermograms of the copolymers are shown in Figure 3.

**Figure 4:**
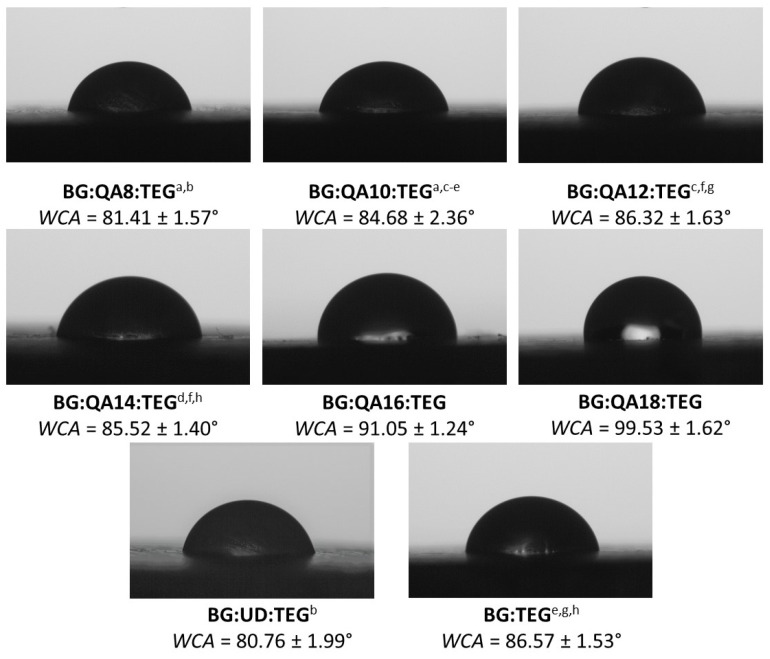
Images captured with the goniometer camera showing droplets of deionized water on the surfaces of the studied copolymers. Lowercase letters indicate statistically insignificant differences (*p* > 0.05, non-parametric Wilcoxon test).

The *WCA* results are presented in Figure 4. The *WCA* of BG:QAm:TEGs ranged from 81.41° to 99.53° and increased as the *Cm* increased. Compared with BG:UD:TEG (*WCA* = 80.76°), all studied BG:QAm:TEGs had higher *WCA* values. Almost all of these differences were statistically significant (*p* ≤ 0.05), except that for BG:QA8:TEG, which was statistically insignificant (*p* > 0.05). Notably, BG:QA8:TEG had a lower *WCA* than BG:TEG (*WCA* = 86.57°), whereas the BG:QAm:TEGs with *Cm* of C10, C12, and C14 had similar *WCA* (*p* > 0.05). The remaining BG:QAm:TEGs had higher *WCA* (*p* ≤ 0.05) than BG:TEG.

**Figure 5:**
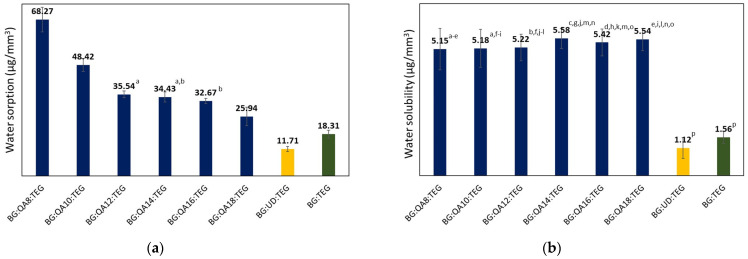
(**a**) Water sorption and (**b**) water solubility of the studied copolymers. Lowercase letters indicate statistically insignificant differences (*p* > 0.05, non-parametric Wilcoxon test).

The *WS* and *SL* results are presented in Figure 5a,b, respectively.

The *WS* of BG:QAm:TEGs ranged from 25.94 to 68.27 µg/mm^3^ and decreased as the *Cm* increased. All BG:QAm:TEGs had higher *WS* (*p* ≤ 0.05) than the reference copolymers (*WS_BG:UD:TEG_* = 11.71 µg/mm^3^, *WS_BG:TEG_* = 18.31 µg/mm^3^). The *SL* values of BG:QAm:TEGs were similar (*p* > 0.05) and ranged from 5.15 to 5.58 µg/mm^3^. Compared with the reference copolymers (*SL_BG:UD:TEG_* = 1.12 µg/mm^3^, *SL_BG:TEG_* = 1.56 µg/mm^3^), all of the studied BG:QAm:TEGs had higher *SL* (*p* ≤ 0.05).

## 3. Discussion

In this study, novel dimethacrylate liquid monomer compositions and their corresponding copolymers consisting of 40 wt.% Bis-GMA, 40 wt.% QAUDMA-m, and 20 wt.% TEGDMA (BG:QAm:TEGs) were prepared, and their physicochemical properties were discussed. It is essential to understand these fundamental properties in order to assess the potential applicability of such copolymers in newly designed dental composite matrices; however, they are rarely studied. 

The organic matrix in a dental composite is responsible for the proper functioning and longevity of dental restoration materials. This matrix sticks the filler particles together, transfers stress to those particles, and gives shape to the restoration. Therefore, the matrix should have adequate physicochemical properties and should be stable over the entire desired service time and intraoral temperature range. 

### 3.1. Properties of Liquid Monomer Compositions

The *RI* defines the optical properties of a composite matrix, which should provide similar transparency to enamel [33]. All of the prepared BG:QAm:TEGs met the requirements for dental materials, according to which the *RI* should be within the range from 1.46 to 1.55 [34]. These results suggest that all tested BG:QAm:TEGs have suitable aesthetic properties. 

The *Tg_m_* of BG:QAm:TEGs ranging from −52.22 to −42.12 °C indicates that all the monomeric systems exist in a liquid state at working temperatures. The *Tg_m_* values were analyzed to assess the molecular mobility of liquid monomers; the higher the *Tg_m_*, the lower the molecular mobility [35]. The lowest *Tg_m_* was recorded for BG:QA18:TEG, which had the highest *Cm*; its *Tg_m_* value was 4.23 °C higher than that of BG:TEG and 7.33 °C lower than that of BG:UD:TEG. In contrast, BG:QA8:TEG, which had the shortest *Cm*, was characterized by the highest *Tg_m_*; its *Tg_m_* value was 14.33 °C and 2.77 °C higher than those of BG:TEG and BG:UD:TEG, respectively. In general, all of the studied BG:QAm:TEGs were characterized by higher *Tg_m_* values than BG:TEG (Figure 2). Relative to the BG:UD:TEG reference, BG:QA8:TEG had a higher *Tg_m_*, BG:QA10:TEG had a similar *Tg_m_*, and the remaining BG:QAm:TEGs had lower *Tg_m_*. These results suggest that the prepared BG:QAm:TEG monomeric systems had lower molecular mobility than BG:TEG. Accordingly, relative to the BG:UD:TEG reference, BG:QA8:TEG showed lower molecular mobility, BG:QA10:TEG showed similar molecular mobility, and the remaining BG:QAm:TEGs showed higher molecular mobility. These distinctions can be explained by differences in the character and the resulting strength of intermolecular interactions between the monomer molecules. Among the two references, BG:UD:TEG had a lower molecular mobility than BG:TEG (i.e., *Tg_m BG:UD:TEG_* > *Tg_m BG:TEG_*) because of the presence of urethane groups in the UDMA monomer. These moieties are willing to form strong hydrogen bonds with the hydroxyl groups of Bis-GMA, which are also stronger than any other hydrogen bonds occurring in systems comprising Bis-GMA, UDMA, and TEGDMA [36]. Owing to the fact that QAUDMA-m molecules can form strong hydrogen bonds with the hydroxyl groups of Bis-GMA as well as UDMA, all of the BG:QAm:TEG liquid monomer compositions were characterized by lower molecular mobility than BG:TEG. Analysis of the *Tg_m_* values further revealed that the *Cm* affects the *Tg_m_*. Specifically, *Tg_m_* decreased (i.e., molecular mobility increased) as *Cm* increased. This relationship can be attributed to the increasing distance between monomer molecules caused by the increasing *Cm*, which weakens the intermolecular interactions. 

### 3.2. Properties of Copolymers

The polymerization shrinkage (*S*) was determined to be a factor influencing the restoration shrinking [37]. This phenomenon results from double-bond polymerization, whereby the van der Waals forces between the monomer molecules are replaced by much stronger covalent bonds. As a result, the entire system shrinks, and marginal gaps are formed between the tooth filling and adjacent tissue [38]. The higher the *S* value, the greater the marginal gap; therefore, the *S* should be as low as possible to enable optimal restoration implantation. In addition, the narrow space between the restoration and the tooth tissue creates an environment that is extremely conducive to bacteria colonization, which can lead to secondary caries or gum inflammation [27].

The *S_t_* can be calculated considering that (i) shrinkage results from the polymerization of the methacrylate group and (ii) the reduced molar volume of methacrylate groups is equal to 22.5 cm^3^/mol [39]. The *S_t_* values of all studied BG:QAm:TEGs were lower than those of the reference copolymers and decreased as *Cm* increased (Table 2). The highest *S_t_* was recorded for BG:QA8:TEG, which was 15.50% and 23.95% lower than those of BG:TEG and BG:UD:TEG, respectively. In contrast, BG:QA18:TEG had the lowest *S_t_*, which was 23.34% and 31.01% lower than those of BG:TEG and BG:UD:TEG, respectively. Thus, all tested BG:QAm:TEG liquid monomer compositions theoretically shrink less than the reference compositions. In addition, increasing the *Cm* reduced the degree of shrinking because increasing the *MW* of the QAUDMA-m decreased the *x_DB_* (Table 1). 

In practice, *S_t_* is always higher than *S_e_* because polymerization is never complete. The *S_e_* was calculated according to the *d_m_* (Table 1) and *d_p_* (Table 2). Similar to *S_t_*, the *S_e_* values of BG:QAm:TEGs were lower than those of the reference copolymers owing to the higher *MW* of BG:QAm:TEG monomer systems. In contrast to *S_t_*, the *S_e_* values of BG:QAm:TEGs increased as *C_m_* increased (Table 2). The highest *S_e_* was recorded for BG:QA18:TEG, which was 20.69% and 23.35% lower than those of BG:TEG and BG:UD:TEG, respectively. The BG:QA8:TEG had the lowest *S_e_*, which was 37.05% and 39.16% lower than those of BG:TEG and BG:UD:TEG, respectively. These relationships can be explained based on the *DC*. Table 2 indicates that the *DC* values of BG:QAm:TEGs increased as *Cm* increased; therefore, *S_e_* also increased.

The *Tg_p_* is related to the stiffness of dimethacrylate polymer networks that create the dental composite matrix; the higher the *Tg_p_*, the stiffer the polymer network [40]. The *Tg_p_* values of BG:QAm:TEGs were lower than those of the reference copolymers and increased as *Cm* increased (Figure 3). The reduction in BG:QAm:TEG stiffness in comparison to the reference copolymers can be attributed to the presence of long *N*-alkyl chains that act as pendant groups and loosen the polymer network structure. The lowest *Tg_p_* was recorded for BG:QA8:TEG, which was 19.25 °C and 13.69 °C lower than that of BG:TEG and BG:UD:TEG, respectively. In contrast, BG:QA18:TEG had the highest *Tg_p_*, which was only 10.65 °C and 5.09 °C lower than that of BG:TEG and BG:UD:TEG, respectively. Considering the target applications of these materials (dental restorations), the *Tg_p_* should be higher than the maximum intraoral temperature to ensure that the material will be in a glassy state, with stable mechanical properties over the entire range of working temperatures [28]. For this reason, the *Tg_p_* should not be lower than 40 °C because the temperature of the oral cavity during a fever usually does not exceed 39 °C [41]. Therefore, it can be concluded that the *Cm* did not negatively affect the *Tg_p_* to a significant extent because the *Tg_p_* values of all studied BG:QAm:TEGs were higher than 40 °C. 

The *WCA* was measured to assess the hydrophilicity of the copolymer surfaces. Common dental composite fillers [42,43] and dental tissues [44] are both highly hydrophilic. Therefore, the higher the matrix hydrophilicity, the greater its ability to physically bond with the filler and tissue adjacent to the restoration. The hydrophilicity can be expressed according to the *WCA*, i.e., if *WCA* < 90°, the surface is hydrophilic, and if *WCA* > 90°, the surface is hydrophobic [45]. The *WCA* values of the studied BG:QAm:TEGs increased as *Cm* increased. As shown in Figure 4, increasing the *Cm* from C8 to C14 caused only a slight increase in *WCA*, which remained lower than 90°. Therefore, the surfaces of these polymers could be classified as hydrophilic. Further lengthening the *Cm* to C16 and C18 significantly increased the *WCA*, thereby changing the nature of the surface from hydrophilic to hydrophobic. These results suggested that the main factor influencing the *WCA* was the length of the *N*-alkyl substituents. The trend observed for *WCA* is consistent with published reports, which indicate that the hydrophobic character of the alkyl chains increases with their length [46]. The slower increase in *WCA* among BG:QAm:TEGs with C8 to C14 relative to those with C16 and C18 was attributed to the dominant influence of the hydrophilic quaternary ammonium group [47] in the BG:QAm:TEGs with shorter *Cm*. In the remaining BG:QAm:TEGs, the hydrophobic nature of the *N*-alkyl chains played a key role. The lowest *WCA* was recorded for BG:QA8:TEG, which was 5.96% lower than that of BG:TEG and 0.80% higher than that of BG:UD:TEG. The BG:QA18:TEG was characterized by the highest *WCA*, which was 14.97% and 23.24% higher than that of BG:TEG and BG:UD:TEG, respectively. These results confirm that the surfaces of all studied BG:QAm:TEGs were less hydrophilic than that of BG:UD:TEG. Compared with BG:TEG, only the surfaces of BG:QA16:TEG and BG:QA18:TEG were more hydrophobic, whereas the remaining BG:QAm:TEGs were more hydrophilic.

Dental materials are constantly exposed to moisture, which results in their swelling due to water absorption [30]. This can compensate for the decrease in material volume caused by polymerization [48]. However, excessive *WS* leads to excess swelling, which may deteriorate the restoration’s mechanical properties, and in extreme cases, cause mechanical damage [49]. Therefore, according to the standard, ISO 4049, the *WS* of dental materials should not exceed 40 µg/mm^3^ [29]. The *WS* values of the studied BG:QAm:TEGs were higher than those of the reference copolymers and decreased as *Cm* increased. The lowest *WS* was recorded for BG:QA18:TEG, which was 41.67% and 121.52% higher than that of BG:TEG and BG:UD:TEG, respectively. The BG:QA8:TEG was characterized by the highest *WS*, which was 272.86% and 483.01% higher than that of BG:TEG and BG:UD:TEG, respectively. The BG:QAm:TEG copolymers with *Cm* from C12 to C18 met the requirements for dental materials because their *WS* values were lower than 40 µg/mm^3^. The high *WS* of studied BG:QAm:TEGs can be explained based on several factors. The most influential factor is likely the presence of two quaternary ammonium groups in the QAUDMA-m, which are primed to absorb water [20]. Additionally, as the *MW* of QAUDMA-m increased (i.e., with increasing *Cm*), the molar ratio of QAUDMA-m in the BG:QAm:TEG decreased; therefore, the proportion of hydrophilic quaternary ammonium groups in the BG:QAm:TEGs decreased, thereby reducing the *WS*. The reduction in *WS* as the *Cm* increased can be explained by the increasing hydrophobicity of the longer *N*-alkyl chains [46]. It is also possible that the longer *N*-alkyl chains can shield the positively charged quaternary nitrogen ion, thus limiting its ability to absorb water [50]. 

Leachability of the residual monomers to water (*SL*) is another parameter that should be controlled when considering materials for dental applications. The *SL* should be as low as possible because excessive leaking decreases the restoration’s lifetime and promotes the deterioration of its mechanical properties [31]. Therefore, according to the standard, ISO 4049, the *SL* of dental materials should not exceed 7.5 µg/mm^3^ [29]. The *SL* values of the studied BG:QAm:TEGs were higher than those of the reference copolymers and were unaffected by the *Cm*. The lowest *SL* was recorded for BG:QA8:TEG, which was 230.13% and 359.82% higher than that of BG:TEG and BG:UD:TEG, respectively. In contrast, BG:QA14:TEG had the highest *SL*, which was 257.69% and 398.21% higher than that of BG:TEG and BG:UD:TEG, respectively. However, all of the studied BG:QAm:TEGs met the requirements for dental materials because their *SL* values were lower than 7.5 µg/mm^3^. The significantly higher *SL* of BG:QAm:TEGs can be explained by the presence of quaternary ammonium groups in QAUDMA-m. As previously stated, these groups have a high affinity to water; therefore, they can easily migrate through the polymer network with water. 

## 4. Materials and Methods

### 4.1. Chemicals and Reagents

The QAUDMA-m analogues were synthesized from 2-(methacryloyloxy)ethyl-2-hydroxyethylmethylalkylammonium bromides (QAHAMAs) TMDI (Tokyo Chemical Industry, Tokyo, Japan). The QAHAMAs were synthesized from *N*,*N*-(2-hydroxyethyl)methylaminoethyl methacrylate (HAMA) and alkyl bromides with chain lengths of 8, 10, 12, 14, 16, or 18 carbon atoms (all purchased from Acros Organics, Geel, Belgium). The HAMA was synthesized from methyl methacrylate (MMA; Acros Organics, Geel, Belgium) and *N*-methyldiethanolamine (MDEA; Acros Organics, Geel, Belgium). All syntheses were performed following the procedures described in a previous report [23]. Camphorquinone (CQ), 2-dimethylaminoethyl methacrylate (DMAEMA), Bis-GMA, TEGDMA, and UDMA were purchased from Sigma-Aldrich, St. Louis, MO, USA and used as received.

### 4.2. Sample Preparation

In total, eight compositions of dimethacrylate monomers were prepared, six of which comprised 40 wt.% Bis-GMA, 40 wt.% QAUDMA-m, and 20 wt.% TEGDMA (i.e., BG:QAm:TEGs). For comparison, compositions of 40 wt.% Bis-GMA, 40 wt.% UDMA, 20 wt.% TEGDMA (i.e., BG:UD:TEG), and 60 wt.% Bis-GMA, 40 wt.% TEGDMA (i.e., BG:TEG) were also prepared. 

The homogenous mixtures of monomers were enriched with a photoinitiating system comprising 0.4 wt.% CQ (initiator) and 1 wt.% DMAEMA (accelerator). The mixtures were poured into Teflon disc-like molds (diameter × thickness = 15 mm × 1.5 mm), covered with a PET film, and subjected to irradiation with a UV-Vis lamp (Ultra Vitalux 300, Osram, Munich, Germany, 280–780 nm wavelength range, radiation exitance = 2400 mW/cm^2^) for 1 h at room temperature.

### 4.3. Refractive Index

The *RI* of the liquid monomer compositions was determined according to the standard, ISO 489:1999 [51], using a DR 6100T (Krüss Optronic, Germany) refractometer. Briefly, 2 mL of each liquid monomer composition was placed on the refractometer plate, and the measurement was performed at 20 °C.

### 4.4. Density and Polymerization Shrinkage

The *d_m_* was determined according to the standard, ISO 1675 [52], using a 1 mL pyknometer. The *d_p_* was determined using an analytical balance (XP Balance, Mettler Toledo, Greifensee, Switzerland) equipped with a density determination kit, which operated on the basis of Archimedes’ principle. 

The *S_e_* was calculated using Equation (1),

(1) $$S_{e}\left(\%\right)=\left(1-\frac{d_{m}}{d_{p}}\right)\times 100$$

where *d_m_* is the liquid monomer composition density, and *d_p_* is the copolymer density.

The *S_t_* was calculated using Equation (2),

(2) $$S_{t}\left(\%\right)=\left(\frac{f\times \Delta V\times d_{m}}{MW}\right)\times 100$$

where *f* is the methacrylate functionality (*f* = 2), Δ*V* is the decrease in molar volume attributable to the polymerization of one mole of methacrylate (Δ*V* = 22.5 cm^3^/mol [39]), *d_m_* is the liquid monomer composition density, and *MW* is the liquid monomer composition molecular weight.

### 4.5. Glass Transition Temperature

The *Tg_m_* and *Tg_p_* were determined using a differential scanning calorimeter (DSC 3, Mettler Toledo, Greifensee, Switzerland) according to the standard, ISO 11357-2:2020 [53]. Briefly, 2 mg of sample were placed in a standard aluminum crucible and heated in air within the temperature range from −90 to 200 °C, with a heating rate of 10 K/min.

The *Tg* was taken as the midpoint of the transition region.

### 4.6. Water Contact Angle

The *WCA* was determined using an OCA15EC goniometer (Data Physics, Filderstadt, Germany) via the sessile drop method. Briefly, 4 µL of deionized water were dropped on the surface of disc-shaped copolymer samples (diameter × thickness = 15 mm × 1.5 mm).

### 4.7. Water Sorption and Solubility

The *WS* and *SL* were determined according to the standard, ISO 4049 [29], using disc-shaped copolymer samples (diameter × thickness = 15 mm × 5 mm). 

Initially, the samples were dried at 100 °C in a conditioning oven until they reached a constant weight (*m*_0_), which was usually achieved after 72 h. Then, samples were placed in glass vials containing distilled water and stored for seven days at room temperature. Next, samples were removed from the water, blotted dry, and weighted (*m*_1_). Finally, the samples were once again dried to a constant mass (*m*_2_) at 100 °C in a conditioning oven. Sample weights were measured using an analytical balance (XP Balance, Mettler Toledo, Greifensee, Switzerland) with 0.01 mg accuracy. 

The *WS* and *SL* were calculated using Equations (3) and (4), respectively,

(3) WS (μgmm3)=m1−m0V

(4) SL (μgmm3)=m0−m2V

where *m*_0_ is the initial mass of the dried sample, *m*_1_ is the mass of the swollen sample, *m*_2_ is the mass of the dried sample after storage in water, and *V* is the initial volume of the dried sample. 

### 4.8. Statistical Analysis

The experimental results reported herein were expressed as an average of five measurements and presented with the associated standard deviations (*SD*). The data were analyzed and compared using the non-parametric Wilcoxon test with a significance level (*p*) of 0.05 using Statistica 13.1 software (TIBCO Software Inc., Palo Alto, CA, USA).

## 5. Conclusions

The physicochemical properties of six dimethacrylate-based compositions comprising 40 wt.% Bis-GMA, 40 wt.% QAUDMA-m, 20 wt.% TEGDMA, and their corresponding copolymers mainly depended on the length of the *N*-alkyl substituent. It was observed that: (i) *Tg_m_* decreased with increasing *Cm*, whereas the opposite trend was observed for *Tg_p_*, (ii) *S_e_* increased with increasing *Cm*, (iii) hydrophobicity (determined from *WCA*) increased with increasing *Cm*, and (iv) *WS* decreased with increasing *Cm*. Only *RI* and *SL* were unaffected by the *Cm*. 

In relation to the properties of the reference copolymers, the following conclusions were drawn. All liquid BG:QAm:TEG monomer compositions had suitable *RI* and *Tg_m_*. Moreover, all studied BG:QAm:TEG copolymers were characterized by low *S* values and *Tg_p_* values higher than 40 °C, suggesting that they would have stable mechanical properties over the working temperature range of dental restorations. The surfaces of two of the BG:QAm:TEGs adopted a hydrophobic character (BG:QA16:TEG and BG:QA18:TEG), whereas the remaining BG:QAm:TEGs had hydrophilic surfaces. All of the studied BG:QAm:TEGs had higher *SL* than the reference copolymers; however, the obtained *SL* values did not exclude their use as matrices of dimethacrylate-based dental composites. Only BG:QA8:TEG and BG:QA10:TEG did not fulfill the *WS* requirements because their *WS* values were higher than 40 µg/mm^3^; this feature would prevent their use as matrices for dental composites. 

## Figures and Tables

**Table 1:** The properties of studied monomer compositions: the length of the *N*-alkyl substituent in QAUDMA-m (*Cm*), molecular weight (*MW*), double-bond concentration (*x_DB_*), refractive index (*RI*), and density (*d_m_*). Lowercase letters indicate statistically insignificant differences with a column (*p* > 0.05, non-parametric Wilcoxon test).

Sample Name	*Cm*	*MW* (g/mol)	*x_DB_* (kg/mol)	*RI* ^1^	*d_m_* (g/cm^3^)	
					Average	*SD*
BG:QA8:TEG	C8	528.74	3.78	1.4997	1.154	0.005
BG:QA10:TEG	C10	535.11	3.74	1.4982	1.142	0.006
BG:QA12:TEG	C12	540.95	3.70	1.5128 ^a,b^	1.127	0.005
BG:QA14:TEG	C14	546.33	3.66	1.5129 ^a,c^	1.116 ^a–d^	0.007
BG:QA16:TEG	C16	551.29	3.63	1.5099	1.113 ^a,e–g^	0.010
BG:QA18:TEG	C18	555.89	3.60	1.5097	1.099 ^b,e,h,i^	0.013
BG:UD:TEG	-	389.27	5.14	1.5127 ^b,c^	1.115 ^c,f,h^	0.007
BG:TEG	-	429.39	4.66	1.5048	1.102 ^d,g,i^	0.005

^1^ The standard deviation of *RI* was 0.0001 in each case.

**Table 2:** The properties of studied copolymers: density (*d_p_*), experimental (*S_e_*), and theoretical (*S_t_*) polymerization shrinkages, and degree of conversion (*DC*). Lowercase letters indicate statistically insignificant differences with a column (*p* > 0.05, non-parametric Wilcoxon test).

Sample Name	*d_p_* (g/cm^3^)		*S_e_* (%)		*S_t_* (%)	*DC* [32]
	Average	SD	Average	SD		
BG:QA8:TEG	1.216 ^a^	0.007	5.08	0.40	9.81	0.59
BG:QA10:TEG	1.208 ^b^	0.004	5.48 ^a^	0.37	9.60	0.60
BG:QA12:TEG	1.200 ^c^	0.002	6.07 ^a–d^	0.49	9.38	0.61
BG:QA14:TEG	1.189 ^d^	0.002	6.14 ^b,e,f^	0.41	9.18	0.63
BG:QA16:TEG	1.186 ^d^	0.005	6.24 ^c,e,g^	0.54	9.04	0.66
BG:QA18:TEG	1.174	0.004	6.40 ^d,f,g^	0.48	8.90	0.68
BG:UD:TEG	1.215 ^a^	0.002	8.35 ^h^	0.23	12.90	0.64
BG:TEG	1.206 ^b,c^	0.003	8.07 ^h^	0.80	11.61	0.68

## Data Availability

Data supporting the reported results are available from the authors upon reasonable request.
